# PReS-FINAL-2075: Biologic response modifiers: usage and safety profile from a North Indian pediatric rheumatology centre

**DOI:** 10.1186/1546-0096-11-S2-P87

**Published:** 2013-12-05

**Authors:** M Agarwal, M Jariwala, S Sawhney

**Affiliations:** 1 Department of Pediatrics, Sir Ganga Ram Hospital, New Delhi, India

## Introduction

Biologic Response modifiers (BRMs) are sparingly used in the Indian subcontinent for two main reasons: economic constraints and concern of infections. We have used BRMs since 2006, albeit with stringent screening.There is a dearth of detail re the use and safety of BRMs from this part of the world. This study was undertaken to add to the knowledge about the use of BRMs in children with rheumatic diseases.

## Objectives

1.To study the use of BRMs in a cohort of pediatric rheumatic diseases2.To evaluate their safety over a longitudinal follow up.

## Methods

Demographic, clinical and treatment details along with the screening used and side effects, if any, were retrospectively collected on a predesigned proforma.

## Results

**Demographics**:105 children(61 boys)were given a BRM(Jan'06-May'13). Median age at diagnosis of a rheumatic ds. was 9.8 yrs(1.25-16.6 yrs)&median age at BRM commencement 12 yrs(2.66-18 yrs). Median duration of follow up after commencement of BRM was 29 mths(1-90 mths) **Usage**:**Indications**:JIA was the commonest diagnosis where BRM was used in 93(ERA:44, SJIA:29, poly:13, Oligo:5), 7had SLE, 2had Kawasaki disease. There was 1 patient each of Takayasu, Sarcoidosis&Overlap syndrome. **BRM used**: Etanercept was the commonest&used in 42 children, Infliximab in 35, Tocilizumab in 34, Rituximab in 8 and Abatacept in 3.69 received one BRM,16 were given 2&1child received 3 BRMs consecutively. **Reasons:**Use of BRMs for each ds:*JIA*:Polyarticular course unresponsive to Methotrexate&steroids:43,early hip involvement:23;cervical spine ds.:7;Sacroiliitis unresponsive to NSAIDs:5,midfoot ds:4,persistent systemic features:7,uveitis:4,chr.MAS:1;secondary amyloid:1. *SLE*:Used in7;nephritis:5,hemolytic anemia:1&coronary artery vasculitis:1. *Misc*:2had Kawasaki disease& 1 had Takayasu. **Pre BRM Screening and results**:*Screen*:Included, Mantoux test,Q-gold,Chest X Ray,USG abdomen, Contrast enhanced CT thorax,HIV,HbsAg&HCV antibodies. *Re*sults:All were negative for HIV, HbsAg&HCV antibodies·Chest X ray normal in104;1had previous endobronchialTB·Mantoux test positive in 4: evaluation confirmed latent TB. BRM commenced after 8 weeks. **Follow up**: Considering the exorbitant cost and the self paying nature for a majority of patients, BRMs were used as remission inducing agents. Of a total of 105 children, 93 had JIA: 56 discontinued BRMs of which 34 remained in clinical remission on DMARDs. 20 stopped due to financial constraints, 7 did not respond to the BRM, 5 had a BRM related adverse effect. **Safety**: There were no serious adverse events. All children were premedicated before infusion& monitored for hemodynamic stability. 5 children had side effects for which BRM had to be stopped (worsening of sarcoid rash in 1, new onset of vasculitic rash in 1, persistent neutropenia in 3). 8 children had allergic rhinitis (during Tocilizumab). Dengue fever was diagnosed in 2, Herpes zoster in 2, acute otitis media in 1, acute parotitis in 1, and varicella in 1 on follow up. 2 children had a positive Mantoux after 1 yr of Infliximab though did not have any evidence of TB on detailed screening. There was no mortality in the cohort.

## Conclusion

BRMs can be safely used as remission inducing agents in a country with a high rate of infectious diseases. Up to 1/3 of cohort remained in clinical remission off BRMs over a median follow up of 29 months. There were no major adverse events.

## Disclosure of interest

None declared.

